# Exploring the Relationship between Inhaled Corticosteroid Usage, Asthma Severity, and Sleep-Disordered Breathing: A Systematic Literature Review

**DOI:** 10.3390/arm92040029

**Published:** 2024-08-09

**Authors:** Marco Zaffanello, Giuliana Ferrante, Michele Piazza, Luana Nosetti, Laura Tenero, Giorgio Piacentini

**Affiliations:** 1 Department of Surgery, Dentistry, Pediatrics and Gynecology, University of Verona, 37100 Verona, Italy; giuliana.ferrante@univr.it (G.F.); michele.piazza@univr.it (M.P.); laura.tenero@aovr.veneto.it (L.T.); giorgio.piacentini@univr.it (G.P.); 2 Pediatric Sleep Disorders Center, Division of Pediatrics, “F. Del Ponte” Hospital, University of Insubria, 21100 Varese, Italy; luana.nosetti@uninsubria.it

**Keywords:** asthma, fluticasone propionate, inhaled corticosteroid, obstructive sleep apnea, sleep-disordered breathing

## Abstract

**Highlights:**

**What are the main findings?**

- In adult patients with asthma, inhaled corticosteroids, especially at high doses, appear to increase the risk of sleep apnea;
- In children, inhaled corticosteroids seem to be a less relevant risk factor for sleep apnea than adults.

**What is the implication of the main finding?**

- The interplay between the use of inhaled corticosteroids, the severity of asthma, and the development of sleep apnea is intricate;
- Since these studies’ high overall risk of bias was categorized as high, further studies are needed.

**Abstract:**

(1) Background: Sleep-disordered breathing and asthma are often interrelated. Children and adults with asthma are more susceptible to sleep apnea. Inhaled corticosteroids effectively reduce inflammation and prevent structural changes in the airways. Objective: to explore the existing literature to determine whether inhaled corticosteroids play a role in sleep-disordered breathing in patients with asthma. (2) Methods: We conducted a thorough search of the PubMed, Scopus, and Web of Science databases for English-language articles published up to 12 May 2024. We utilized the ROBINS-E tool to assess the risk of bias. (4) Conclusions: 136 articles were discerned upon conducting the literature search. A total of 13 articles underwent exhaustive full-text scrutiny, resulting in 6 being considered non-relevant. The remaining seven articles, assessed for eligibility, were incorporated into the final analysis. Five studies were identified in adults and two in children. In adult patients, inhaled corticosteroids, especially at high doses, appear to increase the risk of sleep apnea in a dose-dependent manner. Moreover, the properties of inhaled corticosteroids, such as particle size, may impact the risk of developing sleep apnea. In children, the severity of asthma is a key factor affecting the prevalence of sleep apnea, whereas inhaled corticosteroids appear to be a less significant risk factor compared to adults. All of the studies reviewed were classified as having a high risk of bias or some concerns regarding bias. Each study revealed at least one type of bias that raised notable concerns. This research highlights a complex interaction between the use of inhaled corticosteroids, the severity of asthma, and the onset of sleep apnea. Additional research is necessary to investigate these relationships further.

## 1. Introduction

### 1.1. Sleep-Disordered Breathing

Sleep-disordered breathing (SDB) can lead to significant health issues in children and adults [1]. SDB is associated with a variety of detrimental effects, including intermittent hypoxia, oxidative stress, sleep fragmentation, high blood pressure, and heart disease [2,3]. Polysomnography (PSG) is considered the definitive method for diagnosing SDB in children [4]. The obstructive sleep apnea (OSA) severity, a common form of SDB, is quantified using the apnea–hypopnea index (AHI) [5].

#### 1.1.1. Incidence in Children

In children, the incidence of SDB has been on the rise. Parent-reported habitual snoring varies from 1.5% to 6%, while sleep apnea episodes range from 0.2% to 4%. Depending on the combination of symptoms reported by parents on questionnaires, the prevalence of SDB ranges from 4% to 11% [6]. The minimum prevalence rate for pediatric OSAS is around 1% [7]. Diagnosed OSA, based on various diagnostic study criteria, ranges from 1% to 4% [6].

#### 1.1.2. Incidence in Adults

Among 286 eligible adult participants (aged 36.8 ± 11.9 years), the 5-year incidence rate is roughly 7.5% for moderate to severe SDB and 16% (or less) for mild to moderate SDB combined [8]. The prevalence of OSA associated with daytime sleepiness is about 3–7% for adult males and 2–5% for adult females in the general population [6,9]. SDB affects approximately 17% of men and 9% of women aged 50 to 70 [10].

#### 1.1.3. Incidence in Elderly Individuals

A systematic review of the evidence confirms that advancing age, male sex, and a higher body-mass index (BMI) increase the prevalence of OSA. Specifically, the prevalence of OSA in certain elderly populations reached up to 90% in men and 78% in women [11]. In men, OSA (AHI ≥ 10 events/h) was present in 18.1% of individuals aged 61 to 100 years [12]. In women, the prevalence of OSA (AHI ≥ 15 events/h) was 7.0% in the same age group [13].

#### 1.1.4. Treatment of OSA

Regarding treatment, medical therapy with intranasal corticosteroids is more effective in reducing AHI than a placebo [14]. Intranasal corticosteroids manage OSA symptoms by reducing inflammation and nasal congestion [15]. In children with mild OSA, intranasal corticosteroids such as budesonide and fluticasone have been shown to improve symptoms and reduce adenoid size, but they do not entirely resolve OSA. However, the effectiveness of intranasal corticosteroids in managing OSA symptoms is a matter of debate [16]. 

Recent evidence has been insufficient to support the efficacy of intranasal corticosteroids for OSA treatment in children [17]. In a recent randomized, double-blind, placebo-controlled study, it was reported that in children aged 5–12 years with OSAS, intranasal corticosteroids treatment did not result in significant changes in PSG outcomes, neurobehavioral symptoms, or OSA symptoms after 3 and 12 months of treatment [18]. 

Adenotonsillectomy (A&T), where applicable, remains the first-line treatment for adenotonsillar hypertrophy (ATH), and it is associated with significant improvements in SDB in most children, being effective in 70% to 100% of patients [19].

For adults, continuous positive airway pressure (CPAP) is recognized as the primary treatment for OSA [20]. Maxillomandibular advancement (MMA) is a very effective surgical treatment for patients with OSA who struggle to tolerate CPAP and whose condition has not responded to other surgical methods [21]. Mandibular Advancement Splints (MAS) are oral devices that adjust the jaw and tongue position to maintain an open airway. They are typically used for patients with mild to moderate OSA [22]. Surgical treatments, such as uvulopalatopharyngoplasty, may be an option for patients with severe OSA or particular anatomical abnormalities. Reducing weight can markedly lessen the severity of OSA in individuals who are overweight or obese [22,23]. Myofunctional therapy is a specific exercise program that targets the tongue and facial muscles to promote optimal muscle tone and function [23].

### 1.2. Asthma

Asthma is a long-term inflammatory disease affecting the airways in the lungs, marked by repeated bouts of chest tightness, coughing, wheezing, and difficulty breathing [24]. 

The overlapping symptoms of OSA and asthma can complicate the diagnosis of either condition. The nocturnal respiratory symptoms of OSA and asthma are often interrelated, necessitating in-hospital PSG for accurate diagnosis, particularly in pediatric cases [25]. 

#### 1.2.1. Prevalence of Asthma

A study has revealed significant disparities in asthma prevalence. Over a two-decade period, the overall prevalence of asthma has fluctuated. It increased from 8.5% in 2003 to 9.6% in 2009, then decreased to 7.0% in 2019 [26]. Asthma prevalence peaked at 17.8% among children aged 5–9 years and then declined to 6.9% among those aged 45–49, only to gradually increase to 8.1% among those aged 65–69 [27]. Across the American lifespan, those 65 years and older had the most significant increase in current asthma prevalence, from 6.0% in 2001 to 8.1% in 2010 [28].

#### 1.2.2. Treatment of Asthma

Regarding treatment, inhaled corticosteroids (ICS)s) effectively reduce inflammation and prevent structural changes in the airways, thereby preserving lung function over time [29]. The Global Initiative for Asthma (GINA) provides comprehensive guidelines for managing and preventing asthma [30]. GINA recommends that all adults, adolescents, and children with asthma receive ICS-containing therapy. Furthermore, GINA no longer recommends using short-acting beta-agonists (SABA) alone for asthma management without using ICSs [30]. 

### 1.3. Coexistence of SDB and Asthma

#### 1.3.1. SDB in Asthmatic Children

Children with asthma are more susceptible to OSA. A significant 60.4% of children with asthma were found to have OSA, a rate notably higher than the prevalence of OSA in the general pediatric population [31,32]. In children (7.45 ± 3.2 years), the prevalence of OSA in asthma patients was 63.0% [33]. In children (6.58 ± 1.8 years) with poorly controlled asthma, OSA was significantly higher than in the non-asthmatic population (OR: 40.9, 12.9–144.1). About 63% of children with severe asthma also suffer from OSAS [34].

#### 1.3.2. SDB in Asthmatic Adults

Asthmatic patients with OSA are 3.6-times more likely to experience uncontrolled asthma [35,36]. A meta-analysis revealed that the prevalence of OSA in adult patients with asthma was 49.5%. In addition, the odds of prevalence of the OSA were 2.64 [1.76, 3.52]-times higher in asthma patients (48.9 ± 10 years) than in non-asthma patients [33]. Specific research on the impact of SDB in asthmatic elderly individuals is limited [33].

### 1.4. Aims of the Study

Given the prevalence of OSA in patients with asthma, this review aims to explore the existing literature to determine whether ICSs play a role in SDB/OSA in patients with asthma.

## 2. Materials and Methods

We comprehensively searched the Medline PubMed, Scopus, and Web of Science databases for English-language articles indexed from their inception until 12 May 2024. We adjusted Medical Subject Heading (MeSH) terms to search each database, including their combinations and truncated synonyms. The systematic review was not registered.

Keywords for PUBMED, SCOPUS, and WebOfScience were the following: (Inhaled Corticosteroids OR Inhaled Glucocorticoids OR Inhaled Steroids OR Inhaled Fluticasone OR inhaled mometasone OR inhaled budesonide OR beclomethasone) AND (Obstructive Sleep Apnea OR OSA OR Sleep Apnea Syndrome OR Obstructive Sleep Disorder OR sleep apnea OR sleep-disordered breathing OR Obstructive sleep apnea/hypopnea syndrome). See the Appendix A for details.

### 2.1. PICOS Criteria

The PICOS criteria [37] were followed to define the research scope and select the included studies. 

The inclusion criteria involved patients of all ages with a confirmed diagnosis of asthma and SDB, patients with asthma and OSA/SDB/snoring treated with ICSs, patients with both conditions observed before and after treatment, changes in the severity of AHI before and after treatment with ICS, patients with OSA and asthma compared to a control group, observational studies with a control group, studies with temporal perspectives (prospective, observational, cross-sectional, and longitudinal), and studies with unspecified temporal structures.

The exclusion criteria were patients with significant comorbidities, genetic disorders, or other asthma-related medical conditions. Studies that do not provide information on the use of ICSs, patients not explicitly treated with ICSs, asthmatic patients without a specified OSA/SDB/snoring status and/or ICS treatment, patients with significant comorbidities that could affect OSA outcomes in asthmatic patients treated with ICSs, articles not written in English, reviews, case reports, letters, studies without specific outcome measures, and duplicate studies identified through various data sources were excluded.

### 2.2. Data Extraction

To minimize potential errors and interpretive bias, two independent reviewers meticulously examined the data extraction process for each study. In discrepancies between reviewers, a third reviewer was consulted to resolve issues, ensuring the accuracy and consistency of data extraction. The PRISMA flow diagram was employed to document and visualize the selection process of included and excluded studies (https://www.prisma-statement.org/prisma-2020-flow-diagram, accessed on 10 June 2024). The study was not registered.

Evaluation of the Risk of Publication Quality Distortion

We thoroughly examined potential sources of bias that could influence the results of these studies. The sources of bias considered included the following:

Measurement of exposure bias: this bias may occur if the exposure is not accurately measured, such as through self-reported questionnaires;

Selection bias: this type of bias can arise if participants are not selected in a representative manner from the target population;

Post-exposure intervention bias may occur if participants receive interventions after exposure to the interest factor;

Missing data bias: this bias can arise if data are missing from the study, such as when participants drop out;

Measurement of outcome bias: this bias may occur if the outcome is not accurately measured, such as when subjective measures are used;

Reporting bias: this type of bias can occur if researchers selectively report results that support their hypothesis.

As a systematic method to assess the risk of bias in observational epidemiological studies, we utilized the ROBINS-E (Risk of Bias in Non-randomized Studies—of Exposure) tool [38]. The questions in these tools meticulously assessed the methods and results of the studies, providing ratings of “High”, “Some concerns”, or “Low”.

## 3. Results

**Figure 1:**
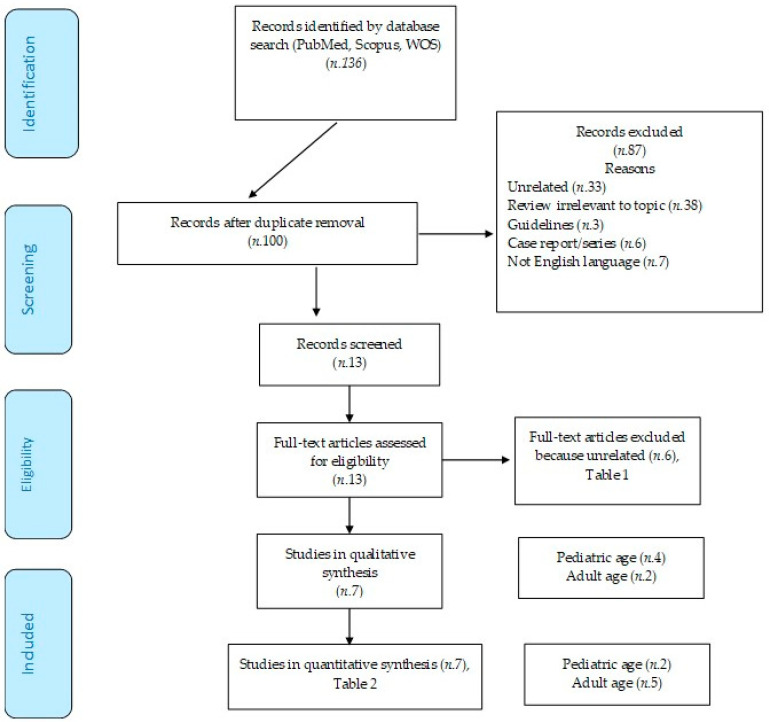
PRISMA flow diagram.

A corpus of 136 articles was discerned upon conducting the literature search, with 36 identified as duplicates. Consequently, 87 studies were excluded: 33 were utterly irrelevant, 38 were immaterial reviews, 3 were guidelines, 6 were non-pertinent case reports or series, and 7 were in languages other than English (Figure 1). A total of 13 articles underwent exhaustive full-text scrutiny, resulting in 6 being considered non-relevant (as encapsulated in the accompanying table). The remaining seven articles, assessed for eligibility, were incorporated into the final analysis (as summarized in the table).

### 3.1. Study Data

The research enumerated in Table 1, omitted from the review, encompasses four pediatric and two adult studies. Bhattacharjee et al. investigated 5942 children who underwent A&T alongside 537 control subjects, aiming to evaluate the impact of A&T on SDB and asthma management. They observed a marked decline in asthma symptoms, ICS usage among children post-A&T, and a simultaneous decrease in SDB [39]. Kheirandish-Gozal et al.’s study involved 92 children with poorly controlled asthma (PCA). Using PSG, the authors assessed the prevalence of OSA in asthmatic children and the effect of A&T on the frequency of asthma exacerbations. They demonstrated a significant improvement in asthma exacerbations and a reduction in the use of rescue β-agonists after A&T treatment [34]. Alfurayh et al. conducted a study on a cohort of 363 children, examining asthma exacerbations in those visiting the emergency department (ED) by analyzing demographic data and comorbidities. They found that most children using ICS did not present nocturnal symptoms, with a low prevalence of OSA [40]. Heatley et al. evaluated the association between intermittent ICS usage and adverse outcomes in asthma patients. Based on medical records from various age groups, they discovered an increased risk of pneumonia and OSA related to cumulative exposure to orally administered corticosteroids (OCSs), with a higher prescription of ICS in patients with frequent OCS use [41]. 

Ferguson et al. conducted a comprehensive analysis of 812 individuals with asthma to investigate the relationships between lower airway dimensions, OSA, and asthma-related parameters with systemic hypertension (HTN). By examining medical histories, spirometry results, and medication regimens, they identified a significant link between high-dose ICS usage and increased HTN risk and a strong correlation between a history of OSA and HTN [42]. Magnoni et al. used questionnaires to study 174 patients in a separate investigation, focusing on epidemiology, risk factors, and treatment strategies. They concluded that combining ICSs with long-acting beta-agonists (LABAs) was the preferred therapeutic approach. At the same time, OSA and obesity emerged as critical comorbidities and risk factors for PCA [43]. 

Although these studies provide relevant insights into asthma and SDB, they diverged from the primary focus of the main investigation. Specifically, the excluded studies targeted different primary outcomes, such as the effects of A&T, the impact of OCSs, or general risk factors like HTN, rather than the direct interplay between ICS and SDB.

**Table 1:** Excluded studies from the review.

First Author, Year	Aim of the Study	Subjects and Methods	Asthma	ICS	OSA or SDB	Conclusions	Reasons of Exclusion
CHILDREN							
Kheirandish-Gozal L, et al., 2011 [34]	Prevalence of OSA in asthmatic PCA children. Effect of A&T on AAE frequency	92/135 children (age 6.58 ± 1.8 years) with PCA; PSG. A&T was performed in the case of OSA. oAHI ≥5/ora TST (n.58)	AAE (n. 92), 3.27 ± 1.13/year AAE: OSA+ (n.58) 3.57 ± 1.37/years OSA− (n.34) 3.12 ± 1.40/years (*p* < 0.05)	β-rescue agonists (4.1 ± 2.4/week) β-Rescue agonists (/week): OSA+: 4.7 ± 2.9 versus OSA− 3.6 ± 2.1 (*p* < 0.04)	β-Rescue agonists (/week): Before A&T OSA+ (No. 35) 4.3 ± 1.8 vs. after A&T 2.1 ± 1.5 (*p* < 0.001) Before PSG OSA− (n.24) 4.2 ± 1.9 vs. after PSG 3.9 ± 2.2 (*p* = NS) AAE (/year): Before A&T OSA+ (No. 35) 4.1 ± 1.3 vs. after A&T 1.8 ± 1.4 (*p* < 0.001) Before PSG OSA− (n.24) 3.5 ± 1.5 vs. after PSG 3.7 ± 1.7 (*p* = NS)	The prevalence of OSA is higher in children with PCA Treatment of OSA with A&T is associated with improvements	Effect of A&T on AAE Frequency in Children With PCA and Associated OSA
Bhattacharjee R, et al., 2014 [39]	A&T+ comparison with controls, SDB, and asthma control	ATH A&T n.5942 (44%) vs. controls n.537 (2%)	AAE decreased from 2243 (30%) pre-A&T to 1566 (2%) post-A&T in children (*p* < 0.0001) Annual reduction in the incidence of hissing by 40.3% in A&T vs. 0% in controls	Reduction in ICS prescription 21.5% A&T vs. −2.0% controls (*p* < 0.001) ICS/LABA −2.2% A&T vs. −20.1% control (*p* < 0.001) Reduction in continuous inhalation for the first hour by 30% in A&T vs. 0% in controls (*p* < 0.001)	Reduction in OSA, snoring, and/or sleep disturbances: A&T n.3603 (27%) vs. control n.1099 (1%)	Children A&T: 30% reduction in AAE 1 year before A&T versus 1 year after 37.9% reduction in ASAs and 35.8% reduction in asthma-related hospitalizations	Efficacy of A&T in Improving Asthma Symptoms and Reducing SDB
Alfurayh MA, et al., 2022 [40]	Exacerbation of bronchial asthma in the ED in a pediatric population	Cohort study: Children in ED due to asthma exacerbation. Data collection: demographics, comorbidities, and asthma-related variables.	Visits to the ED: yes (33.9%) vs. no (66.1%) Of the 123 patients who used steroids, 74% (91) had no nocturnal symptoms (*p* < 0.001)	Of the 363 asthma patients (age 4.9 ± 2.5 years; 68.8% male), 33.9% (n.123) used steroids for asthma	1.9% with FBO (n.7). Number of patients hospitalized with OSA 4.5% (*p* = 0.203).	Association between steroid use in asthmatic patients, number of ED visits, and nocturnal symptoms	Steroid Use in Asthmatic Patients, Number of ED Visits, and Nocturnal Symptoms
Heatley H, et al., 2023 [41]	Intermittent prescribing of OCSs in asthmatic patients and the association with adverse outcomes	Cohort study. Primary Care medical records (ages 4– <12, 12– <18, 18– <65 and ≥65 years) received intermittent OCSs Categories: prescription: single, least frequent (≥90-day range), and frequent (<90-day interval) Controls: patients not treated with OCSs, matched 1:1	Dose–response relationship between cumulative annual exposure to OCSs and risk of adverse outcomes	ICS prescriptions (categorized as 0, 1–3, 4–6, 7–9, 10–12, and ≥13 administration) 12 months prior to initial OCS prescriptions: received 1–2 administrations of SABA and ≤3 of ICSs Proportion of patients receiving ≥3 administrations of SABA and ≥4 of ICSs at baseline increased with more frequent OCS prescriptions Higher number of ICS prescriptions in those who had more frequent OCS prescriptions	Higher risks of adverse outcomes related to OCSs, pneumonia, and OSA	Patients with asthma who received intermittent OCSs have a frequent prescription. Prescribing more frequent OCSs associated with higher risk of adverse outcomes	Association Between Intermittent OCS Prescribing in Asthmatic Patients and Adverse Outcomes, Such as Pneumonia and OSA
ADULTS							
Ferguson S, et al., 2014 [42]	Association between lower airway Caliber, OSA, and other asthma-related factors with HTN	Multicenter study; 812 asthmatics (ages 46 ± 14) OSA scale of the SA-SDQ medical records: HTN, OSA, spirometry, and medications	Subjects with asthma, use of ICSs n.631 (78%): low dose n.189 (23%), medium dose n.235 (29%), and high dose n.207 (25%)	Associations of HTN: Low-dose ICS (OR 0.86, C.I. 0.50–1.45), medium doses (OR 1.1, C.I. 0.75–1.95), and high-dose (OR 2.18, C.I. 1.37–3.48)	Association of HTN with a history of OSA (OR= 5.18, C.I. 3.66–7.32; *p* < 0.0001) and high risk of OSA according to SA-SDQ (OR 5.18 C.I. 3.66–7.32, *p* < 0.001)	Concomitant OSA has been associated with HTN	Association Between OSA and ICSs, with Hypertension in Asthmatic Patients
Magnoni MS, et al., 2017 [43]	How Italian allergists deal with asthma patients	174 questionnaires, 16 questions: Epidemiology, risk factors, therapeutic approaches and adherence to therapy	Follow-up visits at 56.5%, worsening of symptoms for 41%, percentage of visits due to adverse effects of drugs 3%	ICSs combined with LABAs were considered the treatment of choice	Sleep apnea and obesity were assessed as the most critical comorbidities/risk factors of PCA	Recognizing and managing OSA could be key to improving asthma control in patients	Survey or a Questionnaire Exploring How talian Allergists Manage Asthma Patients, Including Their Treatment Approaches and Asthma Management

Legend: AAE, acute asthmatic exacerbations; ACT, asthma control test score; AO, adverse outcomes; ATH, hypertrophy of tonsils and adenoids; A&T, adenotonsillectomy; C.I., confidence interval; ED, emergency department; ESS, Epworth Sleepiness Scale; HTN, systemic hypertension; ICSs, inhaled corticosteroids; LABAs, long-acting beta-agonists; oAHI, obstructive apnea hypopnea index; OCSs, Oral corticosteroids; OSA, obstructive sleep apnea; PCA, poorly controlled asthma; PSA, polysomnography; TST, total sleep time.

The studies in Table 2, included in the analysis, were chosen for their direct relevance to the topic. Five studies were identified in adults and two in children. 

### 3.2. Studies in Adulthood

A survey-based investigation examined 284 asthma patients, averaging 46 ± 13 years (range 18–75 years). The authors discovered a linear and dose-dependent relationship between ICS usage (OR 4.05; 95% C.I. 1.56–10.53) and the risk of OSA, irrespective of asthma severity [44]. Specifically, asthma patients on ICSs, especially at higher doses, showed a significantly increased risk of developing OSA. The risk heightened with increased ICS doses (OR 2.29 for low doses, OR 3.67 for medium doses, and OR 5.43 for high doses) [44]. 

A prospective, single-center, single-group study included 18 asthmatic participants with a mean age of 25.9 ± 6.3 years (range 18–75 years). This research examined the effects of ICSs on upper airway collapsibility (UAW) during sleep and wakefulness in asthmatic subjects. Participants were treated with fluticasone propionate (FP) for 16 weeks. Evaluations included passive critical closing pressure (Pcrit) and nuclear magnetic resonance imaging (MRI) to measure the fat fraction and volume around the upper airways [45]. The results indicated variable changes in the AHI, with some patients improving, others worsening, and some showing no significant changes [45].

A retrospective cohort study analyzed factors linked to habitual snoring and the risk of OSA in a large sample of 38,840 asthmatic patients (mean age 52.8 ± 18.1 years; range 20– ≥65 years) and 155,347 individuals without asthma (mean age 53.3 ± 18.0 years; range 20– ≥65 years). The follow-up duration was around 7 years for asthmatic patients and approximately 6.5 years for the control group [46]. OSA’s adjusted hazard ratio (HR) was 1.87-fold (95% C.I. 1.61–2.17) higher in the asthma cohort than in the non-asthma cohort. Findings indicated that asthmatic patients on ICSs had a heightened risk of developing OSA compared to those not receiving ICS treatment, with an adjusted HR of 1.33 (95% C.I. 1.01–1.76). Additionally, the asthmatic cohort overall exhibited a significantly higher risk of OSA compared to non-asthmatic individuals [46].

A prospective, randomized, and controlled trial investigated the impact of cPAP on asthma control, airway reactivity, daytime sleepiness, and overall health status in asthmatic patients with nocturnal symptoms and OSA. The study included 122 participants with asthma (≥18 years; mean age 50.5 ± 12.0 years), divided into two groups based on the AHI: ≥10 (*n* = 41) and <10 (*n* = 81). Participants were further split into CPAP (*n* = 17) and control (*n* = 20) groups [47]. The results showed no significant differences in AHI changes between the groups. However, AHI was associated with BMI and neck circumference, suggesting that OSA in asthmatic patients may be influenced by anthropometric factors and ICS use [47].

A study investigated the influence of ICSs on the diagnosis of OSA, with a particular focus on the particle size of ICSs. The risk linked to ICSs can differ based on the size of the particles they emit. The size of particles generated via inhalers ranges from less than 1 μm to over 10 μm in diameter. To effectively reach the airways, particles should be smaller than 5 μm. This cohort study included 29,816 asthmatic patients with an average age of 42.8 ± 21.1 years. Evaluations included the asthma control test (ACT) and pulmonary function test (PFT) [48]. Findings revealed that the use of ICSs with standard-size particles was linked to an increased risk of OSA (adjusted odds ratio [aOR] 1.56; 95% C.I. 1.45–1.69), whereas ICS with extra-fine particles (<2 μm) did not significantly elevate the risk (aOR 1.11; 95% C.I. 0.78–1.58). Additionally, asthma control plays a crucial role in the risk of OSA, with a higher probability of OSA diagnosis observed in patients with uncontrolled asthma [48]. 

### 3.3. Studies in Childhood

A prospective observational comparative study examined 108 asthmatic children with an average age of 9.1 ± 3.4 years (range 4 to 18 years). The participants were categorized into groups without SDB (*n* = 76) and with SDB (*n* = 32). The study analyzed the associations between SDB, obesity, and asthma severity at follow-up [49]. Children with severe asthma exhibited a higher frequency of SDB compared to those with mild or moderate asthma. However, no significant correlation was found between SDB and asthma severity in children using high-dose ICS alone or in combination with other medications [49].

A retrospective study investigated the relationships between sleep, obesity, and asthma in urban minority children. This study reviewed the medical records of 443 children with asthma (average age 10.2 ± 4.1 years; range 7–18 years) who underwent PSG. The analysis examined the relationships between spirometry measurements, BMI, and PSG parameters, while accounting for asthma and antiallergic medications [50]. Findings indicated that obese children with asthma had a significant AHI (5.9 ± 12.1 events/h) compared to their normal-weight counterparts (3.1 ± 5.7 events/h; *p* = 0.009). ICS usage was prevalent in both groups but did not directly correlate with AHI [50].

**Table 2:** Included studies from the review.

First Author, Year	Aim of the Study	Subjects and Methods	Inhaled Corticosteroid	Asthma	OSA and SDB	Conclusion
ADULTS						
Teodorescu M, et al., 2009 [44]	Risk Factors Associated with Habitual Snoring and OSA Risk in Asthmatic Patients	Survey of 284 asthmatics (age 46 ± 13 range 18–75 years). N.143/284 (50%) had SDB or met the criteria for high OSA risk. Valutazione SDB: Self-Reported OSA, Symptom, SA-SDQ	Use of ICSs: n.201 (82%): low dose 31, medium dose 87, and high dose 83. No. 65 patients with grade 1 asthma: n.13 (20%) with high doses of ICSs; n.20 (31%) without ICSs. N.77 patients with grade 4 asthma => n.12 (16%) non-use of ICSs; n.43 (56%) high doses of ICSs.	Recent spirometry data collected to assess asthma severity step: predictor of habitual snoring in 244 asthma patients was asthma severity step, aOR 1.22 (95% C.I. 0.94–1.60), *p* = 0.14. Predictor of high-risk OSA in 244 asthma patients was asthma severity step aOR 1.59 (95% C.I. 1.23–2.06), *p* < 0.001.	+129% risk of OSA with low-dose ICSs (OR, 2.29; C.I. 95% = 0.66–7.96); +267% with mid-dose ICSs (OR, 3.67; C.I. 95% = 1.34–10.03); and +443% with high-dose ICSs (OR, 5.43; 95% C.I. = 1.96–15.05) compared to no use of ICSs. Dose-dependent relationship between habitual snoring and ICS dose (overall *p* = 0.004). Dose-dependent relationship between high OSA risk and ICS dose (overall *p* < 0.001).	Increased risk of OSA associated with ICS use. Proportional increase in risk based on the dosage of ICSs used.
Teodorescu M, et al., 2014 [45]	Effects of Orally Inhaled FP on UAW During Sleep and Wakefulness in Asthmatic Subjects	Prospective, single group and center study. Baseline: 18 participants with asthma (age 25.9 ± 6.3 years; range 18–75 years). 16-week ICS (FP) treatment. Asthma duration: 14.4 ± 10.2 years. Pcrit; MRI (fat fraction and volume around the upper airway). Valutazione SDB: Self-Reported OSA Symptom, SA-SDQ	High dose inhaled FP (1760 mcg/day). Dose adherence of FP was 91.2% ± 1.7%.	FEV1% pretreatment 88.8 ± 1.9; post treatment 94.1 ± 0.1 (*p* = 0.001).	AHI baseline (events/h) = 1.2 ± 2.0, improved n.8 (0.51 ± 0.48), unchanged n.8 (1.64 ± 0.79), and worsened n.2 (2.40 ± 2.40). SA-SDQ baseline score = n.18 (21.2 ± 3.9), improved no. 8 (19.38 ± 0.98), unchanged n.8 (22.00 ± 1.40), and worsened No. 2 (25.00 ± 4.00). Pcrit: improved n.8 (−8.16 ± 1.36), unchanged n.8 (−8.51 ± 2.18), and worsened n.2 (−7.35 ± 0.85). Changes in tongue strength with fluticasone inhaled treatment, in the anterior (*p* = 0.02) and posterior (*p* = 0.002) positions.	High-dose FP led to improvements in lung function (FEV1%). Improved Pcrit in some participants. No significant impact on AHI after FP treatment. No reduction in overall AHI. High-dose FP appears to be associated with an increase in fat fraction and total fat volume in surrounding upper airway structures.
Shen TC, et al., 2015 [46]	Factors Associated with Habitual Snoring and SDB Risk in Asthmatic Patients	Retrospective cohort study. With asthma: 38,840 (age 52.8 ± 18.1 years; range 20–≥ 65 years). Asthma-free: 155,347 (age 53.3 ± 18.0 years; range 20–≥ 65 years). Follow-up period: with asthma 6.95 ± 3.33 years; control 6.51 ± 3.44 years. SDB Rating: PSG	OSA risk ratio among asthma patients based on different treatments. ICS 11,214 (15.3 per 1000 persons/year). No ICS 13,792 (10.6 per 1000 persons/year).	aHR +2.51 (95% C.I. (1.61, 2.17) of OSA in the asthmatic cohort compared to control (12.1 vs. 4.84 per 1000 person-years). OSA development during follow-up: aHR +1.87 (95% C.I. = 1.61–2.17) for the asthma cohort compared to the non-asthma cohort.	OSA in asthma patients: Non-steroid aHR 1 (reference). Inhaled steroid aHR 1.33 (95% C.I. 1.01–1.76).	Overall incidence of OSA is higher in the asthmatic cohort than in the control cohort. ICS appears to be associated with an even higher incidence of OSA among asthmatic patients.
Henao MP, et al., 2020 [48]	Effects of ICS on the Diagnosis of OSA, with Sub-Analysis by Particle Size of ICSs.	Cohort study. 29,816 asthmatics (age 42.8 ± 21.1 years). ACT and PFT [A diagnosis of SDB was determined using ICD-9 or ICD-10 codes].	Higher likelihood of OSA in ICS users with standard particle sizes (aOR +1.56, 95% C.I. 1.45–1.69) than in non-users. There was no increased risk of OSA in users of ICSs with extra-fine particles compared to asthmatics who did not use ICS (aOR 1.11, 95% C.I. 0.78–1.58).	Patients with uncontrolled asthma showed a higher likelihood of receiving a diagnosis of OSA. ACT score (aOR +1.60, 95% C.I. 1.32–1.94) among n.1380 uncontrolled asthma versus 3288 controlled asthma. PFT score (aOR +1.45, 95% C.I. 1.19–1.77) among 1229 uncontrolled and 1199 controlled asthma. ICS users were more likely to have OSA, regardless of asthma control (aOR 1.58, 95% C.I. 1.47–1.70).	Probability of having a diagnosis of OSA with normal-sized particle ICSs (OR 1.55, 95% C.I. 1.11–2.16) compared to those with extra-fine particles. Increased odds of having OSA in BMI patients ≥ 25 users of normal-sized-particle ICSs compared to users of extra-fine particles (aOR 1.70, 95% C.I. 1.15–2.50). Increased odds of receiving OSA diagnosis in male BMI ≥ 25 users of normal-sized-particle ICSs compared to extra-fine particles (aOR 2.45, 95% C.I. 1.22–4.93).	Compared to non-users of ICSs, there is an increased risk of OSA among users of ICSs with standard-sized particles. No increased risk of OSA was observed among users of ICSs with extra-fine particles. Patients with PCA showed a higher likelihood of OSA. The association between ICSs and OSA might vary based on asthma control and individual patient characteristics, such as BMI.
Ng SSS, et al., 2018 [47]	cPAP Effect on: Asthma Control, Airway Responsiveness, Daytime Sleepiness, and Health Status in Asthmatic Patients With Nocturnal Symptoms and OSAS	Prospective, randomized controlled trial. Baseline: 122 asthmatic subjects (≥18 years; age 50.5 ± 12.0 years). SDB Rating: PSG Patients with AHI ≥ 10 (*n* = 41). Patients with AHI < 10 (*n* = 81). CPAP group (*n* = 17) and control group (*n* = 20).	Beclomethasone 500 μg or more per day within the last 3 months. Baseline. High-dose inhaled steroids 90.1%. Medium-dose inhaled steroids 9.9%,	Baseline FEV1 (% predetto) 79.2 ± 20.5. No significant difference in the change in the ACT score between n.17 CPAP group 15.9 ± 2.6 vs. n.20 control group 21.7 ± 10.1 (*p* = 0.145).	AHI correlates with BMI (r = 0.255, *p* = 0.008) and neck circumference (r = 0.247, *p* = 0.007). No significant difference in the change in the AHI score between n.17 CPAP group 19.1 ± 11.4 vs. n.20 control group 21.7 ± 10.1 (*p* = 0.474).	Asthma control did not improve significantly despite taking at least a moderate dose of ICSs. This therapy may not be effective in improving asthmatic symptoms in patients with concomitant asthma and OSA.
CHILDREN						
Ross KR, et al., 2012 [49]	Relationships Between Obesity, SDB, and Asthma Severity in Children	Prospective observational study, comparative study. Baseline: 108 (82%) asthmatic children (age 9.1 ± 3.4 years; range 4 to 18 years). Valutazione SDB: overnight finger pulse oximetry monitoring. No SDB (n.76) age 9.3 ± 3.4 years; SDB (n.32) age 8.7 ± 3.3 years. Predicted FEV1%: No SDB 98.7 ± 17.7; with SDB 90.9 ± 17.1. Associations between SDB, obesity, and asthma severity at follow-up.	Severe asthma: children using high-dose ICSs alone or in combination with other drugs. Not severe asthma: low to moderate dose ICS.	Asthma severity at 12-month follow-up: mild/mod (n.79) and severe (n.29). Asthmatic children with BMI z-score = 2 and SDB had a +6.7-fold risk (OR 1.74; 95% C.I.: 25.55) of having severe asthma compared to those without SDB. Children with asthma, BMI z-score 0, and SDB did not have an increased risk (OR +1.40; C.I. 95% 0.31–6.42) of having severe asthma compared to those without SDB.	32 children (29.6%) with SDB. Children with prevalent SDB (OR 4.85, 95% C.I. 1.94–12.10) in severe asthma (55.2%) vs. mild/mod asthma (20.3%, *p* < 0.01). Children with SDB had an OR of 5.02 (95% C.I. 1.88 −13.44) to have severe asthma at follow-up (12 months), after adjustment for BMI z-score (*p* = 0.001).	Children who are asthmatic, obese, and with SDB have a higher risk of severe asthma than those without SDB. Asthmatic, normal-weight, and SDB children using high doses of ICSs alone or in combination with other medications: there was no significant association between SDB and asthma severity.
Conrad LA, et al., 2022 [50]	Associations Between Sleep, Obesity, and Asthma in Urban Minority Children	Retrospective review of medical records; 448 children with asthma (ages 10.2 ± 4.1 years; range 7–18 years) who performed PSG. Association between spirometry variables, BMI, and PSG parameters, adjusting for asthma and anti-allergy medications.	Inhaled steroids: obese asthmatics n.214 (74.1%) and normal weight asthmatics n.125 (81.2%) (*p* = 0.09). [Montelukast: obese asthmatics n.174 (60.2%) versus n.92 (59,7%), *p* = 0,92]. [Nasal steroids: asthmatics obese n.89 (30.8%) versus asthmatics normal weight n.44 (28.6%); *p* = 0.63].	FEV1: Obese asthmatics 83.1 ± 16.5, normal-weight asthmatics 86.4 ± 18.7 (*p* = 0.05). FEF25%–75%: Obese asthmatics 74.8 ± 26.5; normal-weight asthmatics 76.8 ± 28.2 (*p* = 0.4).	289 obese asthmatics 5.9 ± 12.1 versus 154 normal-weight asthmatics 3.1 ± 5.7 (*p* = 0.009).	In obese asthmatic children, both ICSs and montelukast are associated with lower AHI. Neither ICSs nor montelukast are associated with sleep respiratory parameters in children with asthma of normal weight.

Legend: AAE, acute asthma exacerbation; ACT, asthma control test scores; AHI, apnea–hypopnea index; aHR, adjusted HR; aOR, adjusted odds ratio; AT, adenotonsillectomy; ASA, acute status asthmaticus; ARERs, asthma-related emergency room visits; ARHs, asthma-related hospitalizations; BMI, body mass index; C.I., confidence interval; CPAP, continuous positive airway pressure; ER, emergency room; FP, fluticasone propionate; GER, gastroesophageal reflux; GINA, Global Initiative for Asthma 2020; HR, hazard ratio; HTN, systemic hypertension; ICSs, inhaled corticosteroids; LABAs, long-acting beta agonists; MRI, nuclear magnetic resonance; OCSs, Oral Corticosteroids; OSAS, obstructive sleep apnea syndrome; PCA, poorly controlled asthma; Pcrit, passive critical closing pressure; PFT, pulmonary function test; PSG, polysomnography; SABA, beta2-agonisti short-acting; SA-SDQ, Sleep Disorders Questionnaire; SDB, sleep-disordered breathing; UAW, upper airway collapsibility.

**Figure 2:**
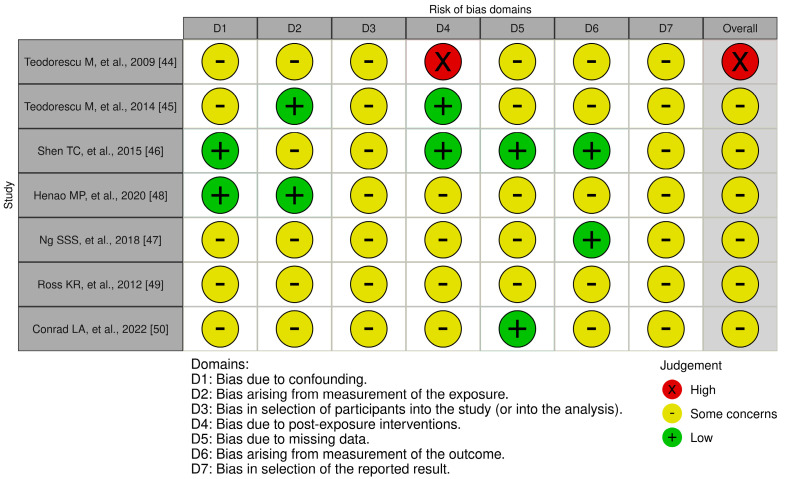
Risk-of-bias plots ROBINS-E [44,45,46,47,48,49,50].

Figure 2 illustrates the results regarding three distinct levels of bias, categorized as “Low” risk, “Some concerns”, and “High” risk. The assessment of bias resulting from confounding revealed that five studies are problematic [44,45,47,49,50]. Bias resulting from exposure measurement was considered problematic or at high risk in five studies [44,46,47,49,50]. Bias in participant selection was also identified as problematic in all studies [45,46,47,48,49,50]. Bias related to post-exposure intervention was found problematic or with increased risk in four studies [47,48,49,50]. Furthermore, bias due to missing data emerged as a significant concern, with a high risk identified in five of the studies [44,45]. Moreover, outcome measurement bias was considered problematic or high risk in five studies [44,45,48]. Reporting outcome selection bias was seen as problematic in 100% of the studies [44]. In summary, all the studies analyzed were categorized as having a high risk of bias or some concerns. Each study identified at least one significant form of bias.

## 4. Discussion

This systematic review showed a linear and dose-dependent relationship between the use of ICSs and the risk of developing OSA in asthma patients, regardless of asthma severity [44]. Specifically, the risk increase is more significant with higher doses of ICSs [44]. Prospective [45] and retrospective [46] studies indicate that ICS use is associated with a high risk of OSA, with anthropometric factors such as BMI and neck circumference further influencing this risk [47]. Standard ICS particles increase the risk of OSA, whereas extra-fine particles do not significantly increase the risk [48]. Implementing routine screening for OSA in patients prescribed higher doses of ICSs, especially those with elevated BMI or neck circumference, could facilitate early detection and intervention.

Additionally, ICSs do not appear to have a direct and significant impact on OSA in children with asthma, but obesity plays a more relevant role in influencing SDB in this population [49,50]. Further research is needed to elucidate the relationship between ICS use and OSA in pediatric populations, particularly in the context of obesity and other comorbid conditions. However, the study results present various significant biases, which require caution in interpreting the conclusions.

The potential mechanism underlying the escalation of OSA or SDB in pediatric and adult asthmatic populations may stem from multiple factors. These include nasal obstruction and bronchoconstriction, leading to increased lower airway resistance and the upper airways’ collapsibility during sleep [51]. Increases in body weight and fat deposits in the upper airway region have also been implicated [52]. The incidence of OSA is generally higher in the asthmatic population compared to non-asthmatics, and this incidence is further elevated in asthmatic patients treated with ICSs [46]. Treatment with FP has been observed to induce notable alterations in the structure of tongue muscles, evident by an augmentation in the percentage of laminin-stained areas across various muscle groups [53]. It is noteworthy that ICS usage can elicit localized effects on upper airway dilators, potentially increasing the susceptibility to OSA, akin to the mechanisms underlying the heightened risk of dysphonia in ICS users [54]. In adults, ICS usage has been linked to an increased risk of OSA, with the risk proportionally rising with higher ICS dosages [44]. Moreover, ICS formulations with larger particle sizes may prompt more pronounced pharyngeal adverse effects than smaller ones [48]. While standard-particle-size ICSs are associated with a greater risk of developing OSA, extra-fine-particle ICSs do not significantly elevate this risk [48]. The size of particles in ICSs emerges as a critical determinant in the context of OSA, with users of extra-fine-particle ICSs exhibiting no significant increase in OSA risk compared to non-users, unlike those using standard particle sizes [48]. This underscores the potential impact of particle size on adverse outcomes, including OSA development, suggesting the opportunity to consider ICS particle size in asthma treatment to mitigate OSA risk in susceptible individuals [48]. Indeed, extra-fine-particle ICSs (<2 μm) disperse more uniformly throughout the lower airways in contrast to larger particle sizes (>5 μm), with larger particles tending to deposit more in the upper airways [48]. 

Prolonged therapy with ICSs has been associated with modulations in hormone secretion patterns, particularly affecting growth hormone release, which may lead to metabolic and cardiovascular complications, thereby exacerbating the impact of OSA [55]. Despite some indications, research findings do not unequivocally support the notion that high-dose FP improves lung function without significantly reducing AHI in asthma patients [45]. Furthermore, it is worth pointing out that high-dose FP may not consistently lower AHI below a clinically significant threshold in all adult patients [56]. Notably, use of ICSs exhibits a dose-dependent relationship with the risk of OSA [44]. The use of FP did not significantly affect the AHI. However, high-dose FP usage appears to be linked to an increased fat fraction and total fat volume in the structures surrounding the upper airways [45]. In obese asthmatic children, the use of ICSs and montelukast is associated with a reduced AHI, although neither ICS nor montelukast significantly impacts sleep respiratory parameters in children with asthma who have normal weight [50].

Asthmatic patients undergoing ICS treatment face an elevated likelihood of OSA development [46]. This risk is particularly pronounced in cases of PCA. OSA exerts an influence on airway inflammation within asthmatic airways and correlates with inadequate asthma control. Although the precise interplay between these conditions remains elusive, inflammation should be recognized as a significant contributing factor [25]. The inflammatory infiltration of the upper airways in asthma and increased fat accumulation in pharyngeal walls due to ICS usage results in a diminished cross-sectional diameter of the upper airways, likely impacting pharyngeal muscle function [25]. Consequently, asthma and its pharmacological treatments can augment the collapsibility of the upper airways, thereby contributing to OSA development and exacerbation [57]. 

Asthma prevalence is influenced by several key factors affecting both children and adults. During childhood, asthma is more frequently observed in males. However, this pattern shifts in adolescence, with females experiencing a higher incidence of asthma that persists into adulthood [58]. Asthma often begins in childhood but can occur at any time throughout life [58]. Higher BMI is associated with adult-onset asthma [59,60]. Atopy is a significant risk factor for childhood asthma and young adult-onset asthma, but not for adult asthma [59]. A family history of asthma is a risk factor for childhood asthma but not adult-onset asthma [59]. Finally, a higher comorbidity burden is associated with persistent severe adult asthma [59]. 

In children, obesity and SDB heighten the risk of severe asthma. Obese asthmatic subjects tend to utilize all classes of asthma medications more frequently and particularly higher doses of ICSs compared to their normal-weight counterparts [61]. Moreover, obesity and SDB escalation amplify the risk of severe asthma in children, particularly in conjunction with elevated ICS doses [49]. The correlation between ICSs and nocturnal respiratory parameters varies depending on BMI [50].

Clinical studies have evidenced the positive impact of CPAP on asthma outcomes in patients concurrently experiencing OSA [62]. CPAP, advocated as the primary treatment for OSA, can potentially ameliorate asthma symptoms in adults [63]. As the frontline intervention for OSAS, CPAP therapy might modulate airway smooth muscle function and enhance asthma management in individuals with both conditions, particularly demonstrating efficacy in severe OSA cases or those with PCA [64]. Consequently, in patients with comorbid asthma and OSA, the effectiveness of ICS therapy in alleviating asthma symptoms might be limited [47]. Notably, asthma control did not show marked improvement with CPAP, even when administering moderate doses of ICSs, suggesting that CPAP might not be effective in alleviating asthma symptoms in patients with asthma and OSA [47].

This review’s limitations encompass the scarcity of available studies and the heterogeneity of research findings from pediatric and adult populations, potentially affecting the uniformity and comparability of results. Disparities in OSA definitions, etiology, and treatments between adults (primarily addressing obesity and CPAP therapy) and pediatrics (concerning adenotonsillar hypertrophy and adenotonsillectomy) further complicate the analysis [47]. Moreover, the overall quality of existing studies is deemed suboptimal.

To date, no research has investigated the relationship between ICS use and OSA in individuals with asthma. Further research is needed to identify the factors contributing to the increased prevalence of OSA among asthma patients, particularly those with severe asthma. Exploring the potential variances in the interaction between ICS and OSA among pediatric and adult patients, likely influenced by distinct OSA etiologies, may shed light on such intricate relationships and guide more effective management strategies for patients affected by asthma and OSA.

## 5. Conclusions

In adult patients, using ICSs, especially at high doses, appears to increase the risk of OSA in a dose-dependent manner. Additionally, the characteristics of ICSs, such as inhaled particle size, may influence the risk of OSA. Implementing routine screening for OSA in patients prescribed higher doses of ICSs, particularly those with elevated BMI or neck circumference, could facilitate early detection and intervention. In children, the severity of asthma plays a crucial role in influencing the prevalence of SDB, whereas the use of ICS seems to be a less relevant risk factor compared to adults. Further research is needed to elucidate the relationship between ICS use and OSA across different age groups, particularly in the context of obesity and comorbid conditions. These studies suggest a complex relationship between ICS use, asthma severity, and the onset of SDB. Additional research is required.

## Data Availability

Data sharing not applicable.

## Appendix A

PUBMED: (Inhaled Corticosteroids [Title/Abstract] OR Inhaled Glucocorticoids [Title/Abstract] OR Inhaled Steroids [Title/Abstract] OR Inhaled Fluticasone [Title/Abstract] OR inhaled mometasone [Title/Abstract] OR ciclesonide [Title/Abstract] OR inhaled budesonide [Title/Abstract] OR beclomethasone [Title/Abstract]) AND (Obstructive Sleep Apnea [Title/Abstract] OR OSA [Title/Abstract] OR Sleep Apnea Syndrome [Title/Abstract] OR Obstructive Sleep Disorder [Title/Abstract] OR sleep apnea [Title/Abstract] OR sleep-disordered breathing [Title/Abstract] OR Obstructive sleep apnea/hypopnea syndrome [Title/Abstract])

SCOPUS: TITLE-ABS-KEY (“Inhaled Corticosteroids” OR “Inhaled Glucocorticoids” OR “Inhaled Steroids” OR “Inhaled Fluticasone” OR “inhaled mometasone” OR “inhaled ciclesonide” OR “inhaled budesonide” OR “inhaled beclomethasone”) AND TITLE-ABS-KEY (“Obstructive Sleep Apnea” OR OSA OR “Sleep Apnoea Syndrome” OR “Obstructive Sleep Disorder” OR “sleep apnea” OR “sleep-disordered breathing” OR “Obstructive sleep apnea/hypopnea syndrome”)

WEBOFSCIENCE: AB = (“Inhaled Corticosteroids” OR “Inhaled Glucocorticoids” OR “Inhaled Steroids” OR “inhaled fluticasone” OR “inhaled mometasone” OR “inhaled ciclesonide” OR “inhaled budesonide” OR “inhaled beclomethasone”) AND AB = (“Obstructive Sleep Apnea” OR OSA OR “Sleep Apnea Syndrome” OR “Obstructive Sleep Disorder” OR “sleep apnea” OR “sleep-disordered breathing” OR “Obstructive sleep apnea/hypopnea syndrome”)
